# Density-Dependent Cladogenesis in Birds

**DOI:** 10.1371/journal.pbio.0060071

**Published:** 2008-03-25

**Authors:** Albert B Phillimore, Trevor D Price

**Affiliations:** 1 Natural Environment Research Council Centre for Population Biology and Division of Biology, Imperial College London, Ascot, Berkshire, United Kingdom; 2 Department of Ecology and Evolution, University of Chicago, Chicago, Illinois, United States of America; University of Edinburgh, United Kingdom

## Abstract

A characteristic signature of adaptive radiation is a slowing of the rate of speciation toward the present. On the basis of molecular phylogenies, studies of single clades have frequently found evidence for a slowdown in diversification rate and have interpreted this as evidence for density dependent speciation. However, we demonstrated via simulation that large clades are expected to show stronger slowdowns than small clades, even if the probability of speciation and extinction remains constant through time. This is a consequence of exponential growth: clades, which, by chance, diversify at above the average rate early in their history, will tend to be large. They will also tend to regress back to the average diversification rate later on, and therefore show a slowdown. We conducted a meta-analysis of the distribution of speciation events through time, focusing on sequence-based phylogenies for 45 clades of birds. Thirteen of the 23 clades (57%) that include more than 20 species show significant slowdowns. The high frequency of slowdowns observed in large clades is even more extreme than expected under a purely stochastic constant-rate model, but is consistent with the adaptive radiation model. Taken together, our data strongly support a model of density-dependent speciation in birds, whereby speciation slows as ecological opportunities and geographical space place limits on clade growth.

## Author Summary

It is probable that the number of species that a given region can support is limited; however, it is unclear whether the limit is approached sufficiently in nature such that the rate at which new species form slows down. Using the pattern of phylogenetic branching, a technique that estimates evolutionary relationships based on molecular data, we demonstrate that in large clades of birds, there is a decrease in the per-lineage probability of speciation as the number of species in the clade increase. We also show that this pattern can arise even if speciation and extinction occur randomly through time. This is because large clades are likely, by chance, to have rapidly speciated early in their history, and will relax back to the average speciation rate later on. We account for this effect, and we still find evidence that, as a clade grows to large size, the per-lineage probability of speciation declines. These results strongly suggest that speciation rates are slowed as environments fill up with competitors.

## Abstract 3

Molecular evidence provides strong evidence that speciation rates slow down through time.

## Introduction

Patterns of speciation vary across both time and space. The changing time course of speciation has been emphasized in the context of adaptive radiations, where it is proposed that a diversity of unexploited resources stimulates a burst of speciation, with speciation slowing down as niches become filled [1–3]. This is for two complementary reasons. First, Mayr [4] noted that speciation may be more difficult in an environment full of competitors, because populations find it more difficult to persist in new locations, which is an essential requirement for populations to differentiate to the level of full species [5]. Second, Rice and Hostert [6] and Schluter [5,7] suggested that reproductive isolation between populations should evolve more quickly early in an adaptive radiation rather than later, because divergent selection pressures are stronger early on. In the fossil record, rates of morphological evolution are clearly episodic, and this is some of the strongest evidence for the process of adaptive radiation [8]. However, because speciation and lineage splitting can occur with little morphological evolution [8,9], the question of whether speciation rates vary through time is best assessed using reconstructed phylogenies [10].

A large number of molecular phylogenies are now available and, when they are calibrated in terms of time, they often show a signature of a decrease in speciation rates toward the present. The evidence comes from a graph of the logarithm of the number of lineages present in the phylogeny against time, referred to as a lineage-through-time plot [10–12]. In a pure birth (or Yule) model [13], with a constant probability of speciation through time and no extinction, the expectation of the lineage-through-time plot is a straight line. In fact, in many studies, as one nears the present, fewer lineages than expected accumulate [11,12,14–22]. Such slowdowns have often been interpreted in terms of adaptive radiation (e.g., [11,17,19,21]). In such cases, the combination of geographical boundaries limiting clade distributions and restricted availability of ecological niches leads to a slowing of speciation rates as species accumulate [23–25].

Although adaptive radiation models predict a slowdown of speciation rate as clades grow large, such a pattern can emerge from simple stochastic models of constant speciation and extinction probabilities. This is because large clades are produced when, by chance, multiple speciation events have happened early during diversification, and small clades are produced when, by chance, few speciation events have happened early. As time proceeds and lineages accumulate, both large and small clades are likely to regress back to the universal average speciation rate, thus generating a slowdown for large clades and a speedup for small clades. We simulated tree growth under pure birth [13] and birth–death [26] models to assess the magnitude of this effect, and showed that the result is generally an inflated type 1 error, which is further exacerbated because researchers tend to study large clades.

We conducted a meta-analysis of 45 phylogenetic reconstructions for clades of birds (at or around the genus level) totaling approximately 1,350 species (depending on how species are defined). We adopt the widely used γ test statistic to test for slowdowns in speciation rate [12]. This statistic relates to the distribution of internode distances through time, and under the pure birth model follows a standard normal distribution with a mean of 0. A γ value less than −1.645 rejects the pure-birth model under a one-tailed test and provides support (α *=* 0.05) for the alternative hypothesis of slowdown. In practice, this is likely to be a conservative test, because the effect of constant extinction rates is to produce the appearance of a speedup in diversification rates on the reconstructed phylogeny (i.e., with constant birth and death rates, the expected value of γ is positive [12]). Across 45 phylogenies, we estimate an average γ value that is significantly less than zero, and this is consistent with a decline in speciation rates through time to half (or less) of the initial rates. Although the clades we focus on may be a nonrandom selection with a probable bias toward large young clades, the frequency of significant slowdowns that we observe in large clades is significantly greater than expected under constant rate models. Results, therefore, provide general support for density-dependent speciation.

## Results

### Simulations

**Figure 1:**
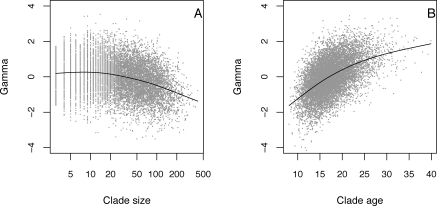
The Relationship between γ and Clade Size and Age from Pure Birth Simulations Plot of the γ statistic versus (A) clade size and (B) clade age in 10,000 simulated datasets of the pure birth model. Clade size simulations were run for 18 time units and clade age simulations were run until clades contained 50 species (both using a birth rate of 0.2). The solid line illustrates the relationship between γ and (A) clade size and (B) clade age, fitted using a cubic smoothing spline to capture non-linearity.

Our simulations showed that even under a pure birth model, a negative correlation of γ with clade size is expected (Figure 1A and Table S1). This is because small clades (e.g., clades containing four species) are biased toward those that began speciating at a slow (below average) rate. Conversely, large clades (e.g., clades of size > 100) are biased toward those that began speciating at a fast (above average) rate. Intermediate sized clades consist of a mixture of lineages that showed rapid and slow rates of early diversification and therefore possess a symmetrical distribution of γ values centered on 0. The average γ value estimated for each set of constant rate parameters tended to be less than 0, due to the smallest clades (i.e., those that should show a strong speed-up) having too few species to permit calculation of γ) (Table S1). The negative correlation between γ and clade size held when the birth rate was allowed to vary between different simulations.

When extinction was added, so long as the death rate, *d*, remained low (<0.25 × birth rate, *b*), the negative association between clade size and γ remained, albeit with a reduced correlation coefficient, when compared to the pure birth model. When *d* = *b* there was no correlation between clade size and γ (Table S1). In the *d* = *b* scenario only those lineages that diversified rapidly initially had surviving members at the end of the simulations [25], with the result that extinction eroded variation in γ.

We conducted two further sets of simulations. First, when we studied clades of the same size, we found that clade age was positively correlated with γ, *r* = 0.52. Thus, younger lineages showed more evidence for slowdowns (Figure 1B). Second, under a constant rate birth-death scenario the strength of the correlation increased to *r* = 0.58 when *b* = 0.25*d*, and *r* = 0.82 when *d* = *b.* The explanation for this positive correlation is similar to that underpinning the negative relationship between clade size and γ. Young clades are typically those that diversified fastest initially before slowing down to the average rate. Older clades are typically those that diversified slowly at first before accelerating up to the average rate.

### Meta-Analysis

In the dataset of 45 bird clades (Table 1), the mean γ = −0.98 ± 0.20 standard error was significantly different from 0 (*t* = 4.89, *p* < 0.001). A third of all clades had a γ ≤ −1.645, which suggests that slowdowns are widespread. However, such a conclusion may be biased by the over-representation of large clades in our dataset. Fifty-seven percent (12/21) of clades with more than 15 lineages at 2 Mya had a γ < −1.645. Although we have shown that under constant rate models, larger clades tend to show a slowdown, the high frequency of significant slowdowns that we observed in such large clades exceeded the null expectation (based on χ^2^ tests across a range of birth and death values, *p* always less than 0.01, see Table S1). Thus, we have evidence that at least some clades are showing a deterministic decline in speciation rate toward the present.

**Table 1:**
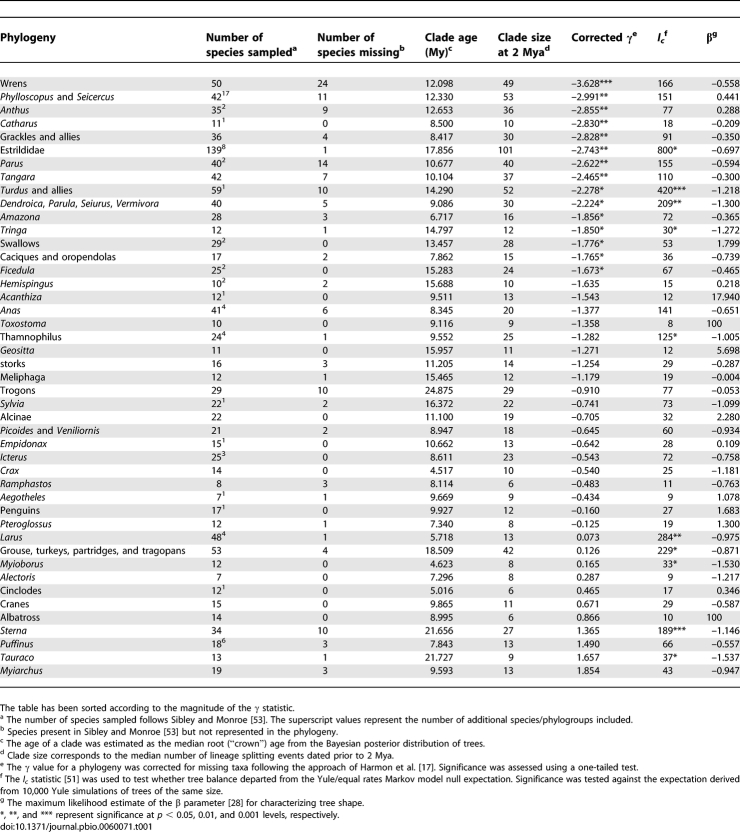
Summary Data for 45 Bird Phylogenies

**Figure 2:**
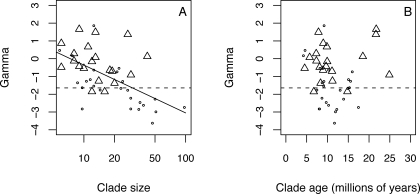
The Observed Relationship between γ and Clade Size and Age across Bird Phylogenies (A) Plot of the γ statistic versus clade size, where clade size is the total number of lineages estimated to be present 2 Mya for 45 bird clades (Table 1). All values below the dashed line are significant at *p* < 0.05. The least squares regression slope is *b* = −1.10 ± 0.24, *p* < 0.001. (B) Plot of the γ statistic versus clade age. Circles and triangles represent passerine and nonpasserine clades, respectively.

The correlation of clade size with γ was highly significant, *r* = −0.58, *p* < 0.001 (Figure 2A). A strong negative correlation is expected whenever there are true slowdowns in the data, because the associated larger sample size of larger clades results in a greater power (more-negative γ) and thus it is difficult to estimate the magnitude of the slowdown. However, simulations described in Protocol S1 suggest that a statistically significant γ value in clades of size 15 requires an average slowing of speciation rate later in the radiation to 10%–50% (or less) of the initial rate.

γ was not correlated with clade age (Figure 1B; *r* = −0.02, *p* = 0.88). When variation in clade size was accounted for using multiple regression, there was a marginally nonsignificant positive correlation between clade age and γ (Table 2). Together, clade age and clade size explained 39% of the total variance in γ.

**Table 2:**
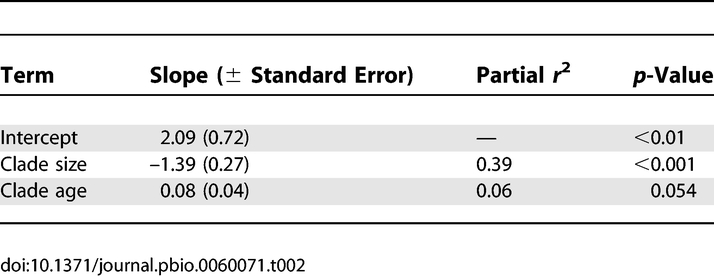
Multiple Regression of γ on Clade size and Clade Age, for *n* = 45 Bird Clades

Ten of the 45 trees were found to be significantly more imbalanced than expected under the equal rates Markov model of tree growth (Table 1). There was some evidence for a general departure from the degree of balance expected under a pure birth model (median β = −0.56), although this departure was less than that reported for a large sample of published trees [27]. After controlling for clade size and age using multiple regression, tree imbalance (estimated using the tree-splitting parameter β [28]) showed a weak positive correlation with γ, *b* = 0.40 ± 0.16, partial *r^2^* = 0.08, *p* < 0.05. This implies that more imbalanced trees have a slightly greater tendency to show speciation slowdowns.

## Discussion

The empirical results provide strong evidence for slowdown in speciation rate in large clades. The magnitude of slowdown seems to be quite large. For example, among the 22 clades with 15 or more lineages at 2 Mya, the median value for γ = −1.77, and the median clade size is 29, consistent with a slowing of speciation rate in the later stages of a radiation to 10%–50% of the initial rate (Protocol S1). Our conclusions are predicated on the assumption that the molecular phylogenies accurately reconstruct the timing of speciation events. In particular, if saturation is present in the molecular data, deep branch lengths will be consistently underestimated, leading to a bias toward a negative γ [29]. Over the time scales of this investigation—and given our use of the complex GTR + I + G model—this seems unlikely to be a problem. Further, if nucleotide saturation is driving patterns, we expect to see greater slowdown in older clades, the opposite of what is observed. Nor is the negative correlation of γ with clade size (Figure 2A) expected to arise as a consequence of nucleotide saturation. Estimates of speciation patterns based on gene trees add error to the estimate of speciation times (e.g., [30]), but this source of error should make slowdowns more difficult to detect, rather than introducing a bias. The observed negative relationship between clade size and γ is not limited to this study. Indeed, two earlier studies based partly on subsets of the data analyzed here reported a similar trend [18,19].

Two predictions regarding temporal patterns of speciation arise from adaptive radiation models [2]. First, clades should show a slowdown in speciation toward the present as niche space is filled. This prediction rests on the assumption that members of a clade, in our case usually a genus, experience competition over niche space. Second, given density-dependent speciation, slowdowns should be particularly evident in large clades [18]. We found strong support for both these predictions. However, the same predictions also arise under a simple model where speciation rates are constant across time (and this also applies to models which allow for low levels of extinction, see Table S1). This is because small clades are likely to have diversified, by chance, slowly early on, and large clades are likely to have diversified quickly early on, both followed by regression to the mean. Thus a bias on the part of researchers toward studying large clades leads to the expectation that those clades that are studied will show the pattern of slowdown. This bias probably affects tests of other questions [25,31]. For example, very few phylogenetic studies have identified an extinction rate greater than zero, which is paradoxical given estimates of extinction derived from fossils [32]. A signal of extinction is identified from reconstructed phylogenies, because, under a constant rate model, extinction leads to an increase in the observed branching rate toward the tips of the reconstructed tree for extant species [10]. Given that we expect to find a decrease in diversification rate toward the present in large young phylogenies (if *b* and *d* are relatively constant through time and *d*/*b* is low), then a bias toward testing for extinction in such large young clades introduces a strong bias against detecting extinction.

Under the adaptive radiation model, a negative correlation between clade age and γ is expected, because rapid initial diversification followed by a slowdown should result in more-pronounced slowdowns in older clades. The reverse is expected under constant rate models: after controlling for clade size, a positive correlation between clade age and γ (i.e., younger clades tend to show the strongest slowdowns) is predicted (Figure 1B). This is because clades that quickly attain a given size are likely to have experienced above-average rates of initial diversification. As lineages accumulate, the overall diversification rate will approach the underlying mean, resulting in slowdowns. Thus our finding of a tendency toward a positive association (albeit marginally nonsignificant) between clade age and γ (Table 2) is more in accord with the constant speciation model than the adaptive radiation model.

Both the adaptive radiation model and constant-rate, stochastic model predict negative γ in large clades, whereas the marginally nonsignificant positive correlation between clade age and γ is more consistent with the constant rate null model. However, constant rate models cannot explain the very high prevalence of significant slowdowns observed in large clades. In particular, our simulations show that if the actual extinction rate across bird lineages over the past 20 million years has approached the speciation rate, then the probability that the strong slowdowns observed in large clades could have arisen under a constant-rate birth–death model becomes vanishingly small. We thus conclude there is strong evidence for density-dependent cladogenesis in large clades.

Speciation rates across a whole clade may slow through time because a few ecologically unusual and/or geographically restricted lineages persist for a long period of time without speciating, even as the rest of the clade continues to follow a constant birth–death model [23]. This should create strong tree imbalance. However, the tree-imbalance parameter we used explains only a small proportion of the variance in γ (partial *r^2^* = 0.08, *n* = 45 clades), and it is likely that slowdowns are the result of more general ecological interactions. For example, in the Old World Leaf Warblers (*Phyllscopus* and *Seicercus*), related sympatric species in the Himalayas are old and occupy different habitats, which are presumed to have arisen in association with mountain building or climate change 8–10 Mya [33]. Even Leaf Warbler allospecies, with abutting geographical ranges, are typically separated by millions of years [33]. We suggest that limited ecological space in this and other groups has restricted the ease of range expansions, and consequently further allopatric speciation. Similarly, Ricklefs [24] found a negative correlation between number of species in a clade and age of the clade across passerine birds, and he interpreted this finding in terms of niche-filling, as we do here. Some alternative explanations for slowdowns have been suggested, including nonrandom extinction [14,34] and episodic appearances of multiple barriers [14,15,20], but these seem less likely to produce such a general pattern.

In conclusion, we find that two factors contribute to the prevalence of slowdowns reported in large phylogenies. First, the strong signal of slowdown supports an adaptive radiation model, where speciation is accelerated in empty environments and slows as niches get filled. Second, speciation events happening randomly within clades through time may also result in the presence of a slowdown in large young clades. Randomness does not mean that speciation is completely unpredictable, but rather that multiple independent causes are likely to contribute [35]. Speciation may be promoted by factors such as occasional extinctions creating new ecological opportunities, appearance of habitat that can be exploited by multiple lineages (rather than a single lineage that rapidly diversifies), the strength of barriers, chance dispersal events, and the occasional evolution of traits within lineages that affect speciation probability. The overall importance of random processes as causes of slowdowns depends on the true extinction rate. If extinction rates are low, the importance of stochastic factors in generating slowdowns may have been underestimated. If, as seems likely, extinction rates approach the speciation rate [36,37], then constant birth–death models on their own cannot explain slowdowns. Instead, our findings of strong slowdowns provide support for nonrandom processes of species diversification through time.

## Materials and Methods

### Simulations.

We used the γ statistic (Equation 1) to assess slowdown [12]. This is based on the cumulative frequency of internode distances (*g*_2_ *–g*_n_) as one counts from the root to the tips of the tree, where *n* equals the number of taxa. Under the pure birth model, this statistic follows a normal distribution with mean = 0 and standard deviation = 1. A negative value of γ indicates that nodes are distributed more toward the root of the phylogeny than expected under the null, implying a slowdown toward the present. One-tailed significance for testing slowdowns at α = 0.05 is γ < −1.645 [12]. Simulations have demonstrated that a decrease in speciation through time can generate significantly negative γ values, but that an increase in extinction through time does not [18].

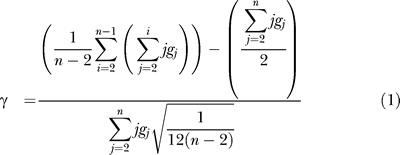
We simulated tree growth under the pure birth model (birth rate *b* = 0.2) for a fixed time duration and calculated the relationship between γ and clade size across 10,000 γ replicates using R code [38] kindly provided by L. Harmon. We repeated this for different durations (19 separate runs, with an arbitrary time duration, assigned integer values between 2 to 20). We also examined variants of this model where extinction (death rates *d* of 0.05 and 0.2) was included and where the birth parameter was allowed to vary among clades (each *b* was obtained from a normal distribution with mean = 0.2 and standard deviation = 0.04). We then examined the relationship between clade age and γ under these constant models, using *Phylogen* v1.1 (available from http://evolve.zoo.ox.ac.uk/) to simulate 10,000 trees of a set size (50 species) under pure birth and birth-death models (*b* = 0.2, *d* = 0, 0.05, and 0.2).

### Data.

We selected bird clades at or about the genus level, for which more than 70% of the species (usually more than 80%) have been sequenced for mitochondrial protein coding genes (Table 1 and Table S2). Typically the amount of sequence available was between 1,000 and 2,000 base pairs, although for a few clades, we only had 600 base pairs. We downloaded sequences from GenBank using *Geneious* [39] and aligned them using *ClustalW* [40] and by eye in the program *MEGA* v3.1 [41]. We reconstructed phylogenetic trees using a relaxed clock Bayesian method [42] implemented in *BEAST v1.4.4* [43]. We set the mean rate of molecular evolution to be 1% per lineage per million years [36], a GTR + I + G model of substitution, and assumed that rate variation among adjacent branches in a tree was uncorrelated and drawn from a log-normal distribution. We used a neighbor-joining model to obtain a prior distribution for the tree, and a pure birth prior on branching rates. We conducted two runs of 5 million generations each and used *Tracer* [44] to assess convergence, that the two runs were sampling from the same posterior distribution, and that the estimated sample size for each parameter was of sufficient size to obtain good parameter estimates (i.e., > 200). There were few cases where the estimated sample size fell below 200 for a single parameter. We used *Tracer* to determine how many burn-in generations to discard, which was always 1 million, except in the Estrildidae (2 million). Using this approach, we were able to obtain a posterior distribution of rooted and dated trees. By sampling every 4,000 generations (6,000 in the Estrildidae), we obtained 2,000 trees from the posterior distribution of the Bayesian runs (1,000 in the Estrildidae). The Bayesian posterior distribution of trees for each of the phylogenies reconstructed from sequences stored in GenBank is available on request from the authors.

We calculated γ across all of the sampled posterior distribution of trees, as follows. First, we counted lineages through time only up to the last bifurcation event prior to 2 Mya. We did this because lineage-splitting events that occur after 2 Mya are not often recorded as different species (especially under the biological species concept [45]), or alternatively over-recorded (as a result of excessive splitting of distinctive populations following a strict application of the phylogenetic species concept, [46,47]). We obtained the median γ across the trees sampled from the Bayesian posterior distribution. All γ estimates were obtained using the *LASER* R library [48,49].

Incomplete sampling can bias estimates of γ [12]. Thus for all phylogenies in which taxon sampling was incomplete, we simulated 2,500 trees of the same size and same number of missing taxa using *PhyloGen* [50]. We obtained a γ value for each simulated tree and adjusted the median γ estimated from our data by subtracting the simulated median and dividing by the standard deviation of the simulated values [17]. This approach assumes that missing taxa are randomly distributed on the tree and also that all missing taxa insert before 2 Mya. The latter assumption may slightly bias the results toward estimating a more-positive γ. However, an alternative approach where we did not correct for missing taxa gave very similar results.

We compared the frequency of significant (γ < −1.645) versus nonsignificant slowdowns observed in large clades (defined as those with more than 15 lineages at 2 Mya) to the null expectation generated under 10,000 constant rate simulations, using a χ^2^ goodness-of-fit test. This comparison was repeated across all of the birth and death parameter space described above (i.e., *b* = 0.2, *d* = 0, 0.05, and 0.2 and simulation duration = 2–20 time units).

Using multiple regression, we tested whether clade size and age were significant predictors of γ. Clade size was calculated as the number of extant lineages in a clade at 2 Mya. Clade age was estimated as the median root age across the posterior distribution of trees.

Pybus and Harvey [12] cautioned that the behavior of the γ statistic on unbalanced phylogenetic trees was unknown. To evaluate the extent to which this was likely to be a problem, we examined whether trees depart from the pure birth/equal rates Markov expectation using Colless' [51] imbalance statistic, *I_c_*. The null expectation for the imbalance statistic across each tree was generated via Monte Carlo simulations of trees of the same size under a Yule (or equal rates Markov) model using the apTreeshape R library [52]. We also calculated the median of the maximum likelihood estimate of the β tree-splitting parameter (examined in the range −2 to 100) for each tree [28] using R code kindly provided by M. Blum [27]. We examined whether the degree of tree imbalance affected γ in a multiple regression in which clade size, clade age, and β were predictors. The β parameter was preferred to the *I_c_* statistic as its expectation under a Yule model is independent of clade size. A β of zero is expected under the Yule null, while β < 0 and > 0 correspond to trees that are more imbalanced or balanced than the Yule expectation.

## Supporting Information

Protocol S1 Simulations of Speciation Slowdowns: The Relationship between Tree Size and γ (42 KB DOC)

Table S1 Results from Pure Birth and Birth–Death Simulations (132 KB DOC)

Table S2 Major Sources of Phylogenetic Information (132 KB DOC)

## Abbreviations

Mya: million years ago
