# Supplementary material for: Density-Dependent Cladogenesis in Birds
Source: PLoS Biol. 2008 Mar 25;6(3):e71. doi: 10.1371/journal.pbio.0060071 (PMC2270327; doi:10.1371/journal.pbio.0060071)
Supplement: Protocol S1 — (42 KB DOC) [file pbio.0060071.sd001.doc]

# Protocol S1: Simulations of speciation slowdowns: the relationship between tree size and γ

Whenever γ is significantly negative we assume that speciation rates have slowed towards the present [1,2]. Because the value of γ depends on both sample size (i.e., clade size) as well as the effect size (i.e., the magnitude of slowdown), in order to obtain a qualitative estimate of the magnitude of slowdown associated with a particular clade size and γ, we used simulations.

# Methods

Trees were grown under a pure birth model with a birth rate, *b*_1_ = 1, starting with a bifurcation using R code kindly provided by David Orme (available from https://r-forge.r-project.org/projects/caic/). Trees were grown for a set amount of time under the initial pure birth conditions (i.e. a rate of 1) and then for a second duration under a pure birth rate = *b*_2_. Across 1,000 simulations we estimated the median tree size and γ statistic [1]. We examined median tree size and γ for the following parameters: i) the total duration of each simulation (2, 2.5, 3, 3.5, 4, 4.5, 5); ii) the value of *b*_2_ (*b*_2_ = 0.1, 0.25, 0.5, 0.75); and iii) the ratio of the time (*t*) in *b*_1_ to time in *b*_2_ (*t* = 1/9, 1, 9).

# Results and Discussion

Across all scenarios a greater degree of slowdown (i.e. a lower value of *b*_2_) led to a more negative γ statistic, as expected (Figure S1 A, B and C). When we simulated the constant rate case (i.e. *b*_2_ = *b*_1_) this typically yielded a γ statistic marginally below 0 for small trees, but γ converged on 0 as tree size increased. When average tree size is small (< 4) the γ statistic cannot be calculated, and these are cases that are more likely to show a positive γ.

When the slow rate, *b*_2_ (*b*_2_ < 1), was applied either half way through or late on in diversification (*t* = 1, *t* = 9) we found a strong negative trend between γ and the median tree size (Figure S1 A and B). This reflects the influence of tree size on the γ test statistic, i.e. the power to reject the null hypothesis. However, we found no relationship between tree size and γ evident when *t* = 1/9 (Figure S1 C). This is probably an artifact of the simulation, whereby the vast majority of internodal distances are sampled under the slower rate.

Interpretation of the γ statistic in terms of magnitude of slowdown thus depends not only on the strength of the slowdown, but when it happened in time, and in general the eventual clade size. However, inspection of the plots in Figure S1A suggest some rough rules of thumb. For example for a clade size of 20, a median γ = -4 represents a strong slowdown, whereby speciation rates are 10% in the second half of the clade’s diversification compared with the first, and a γ = -2 (i.e., close to the level [-1.645] at which one-tailed significance is obtained) still requires a rate in the second half of the clade’s diversification 25% that of the rate in the first half. Indeed, under all our simulation scenarios a slowdown by as much as 50% in the second half of a clade’s diversification would on average not be detectable (i.e. median γ > -1.645) in clades that include < 75 species.


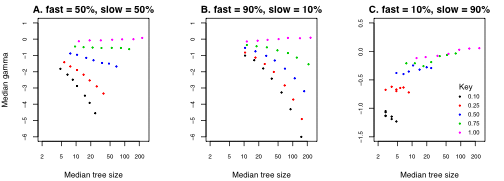


Figure 1. The relationship between tree size and λ under a range of slowdown scenarios. Plots A, B and C differ in the relative duration of the initial rapid Yule rate and the latter slow Yule rate. Note that the of the Y-axis scale differs in plot C. Points are colored to illustrate the affect of differences in rate on λ; the values in the key reflect *b*_2_. Each data point represents the median values obtained from 1000 simulations.

# References

1. Pybus OG, Harvey PH (2000) Testing macro-evolutionary models using incomplete molecular phylogenies. Proc R Soc Lond B 267: 2267-2272.

2. Weir JT (2006) Divergent timing and patterns of species accumulation in lowland and highland neotropical birds. Evolution 60: 842–855.
