# Supplementary material for: Density-Dependent Cladogenesis in Birds
Source: PLoS Biol. 2008 Mar 25;6(3):e71. doi: 10.1371/journal.pbio.0060071 (PMC2270327; doi:10.1371/journal.pbio.0060071)
Supplement: Table S1 — (132 KB DOC) [file pbio.0060071.st001.doc]

Table S1. Results from pure birth and birth-death simulations.

| Model^†^ | Birth-death duration^‡^ | Average clade size^§^ | Clade size ≥ 3 (proportion)^¶^ | Mean γ | Intercept^††^ | Slope^††^ | R^2††^ | Sig. slowdown in large clades (proportion) ^‡‡^ | χ^2§§^ |
| --- | --- | --- | --- | --- | --- | --- | --- | --- | --- |
| b=0.2 | 2 | 0.328 | 0.037 | -0.224 | 0.220 | -0.364 | 0.006 | - | - |
| b=0.2 | 3 | 0.555 | 0.096 | -0.203 | 0.559 | -0.592 | 0.023 | - | - |
| b=0.2 | 4 | 0.799 | 0.166 | -0.250 | 0.430 | -0.493 | 0.025 | - | - |
| b=0.2 | 5 | 1.135 | 0.256 | -0.248 | 0.427 | -0.459 | 0.030 | - | - |
| b=0.2 | 6 | 1.473 | 0.342 | -0.268 | 0.361 | -0.399 | 0.032 | 0.250 | 11.571*** |
| b=0.2 | 7 | 1.952 | 0.425 | -0.216 | 0.617 | -0.496 | 0.058 | 0.253 | 11.267*** |
| b=0.2 | 8 | 2.525 | 0.519 | -0.231 | 0.444 | -0.379 | 0.041 | 0.194 | 17.731*** |
| b=0.2 | 9 | 3.113 | 0.583 | -0.199 | 0.611 | -0.427 | 0.063 | 0.170 | 22.190*** |
| b=0.2 | 10 | 3.908 | 0.646 | -0.195 | 0.538 | -0.364 | 0.055 | 0.130 | 33.398*** |
| b=0.2 | 11 | 4.925 | 0.704 | -0.210 | 0.546 | -0.350 | 0.062 | 0.121 | 36.961*** |
| b=0.2 | 12 | 6.106 | 0.755 | -0.194 | 0.620 | -0.354 | 0.069 | 0.118 | 38.270*** |
| b=0.2 | 13 | 7.366 | 0.791 | -0.181 | 0.620 | -0.330 | 0.068 | 0.109 | 42.638*** |
| b=0.2 | 14 | 9.214 | 0.832 | -0.156 | 0.648 | -0.311 | 0.067 | 0.095 | 51.092*** |
| b=0.2 | 15 | 11.234 | 0.860 | -0.144 | 0.639 | -0.287 | 0.066 | 0.075 | 68.688*** |
| b=0.2 | 16 | 13.688 | 0.879 | -0.121 | 0.676 | -0.275 | 0.065 | 0.074 | 69.818*** |
| b=0.2 | 17 | 16.766 | 0.898 | -0.099 | 0.806 | -0.295 | 0.082 | 0.069 | 75.965*** |
| b=0.2 | 18 | 20.721 | 0.922 | -0.090 | 0.806 | -0.278 | 0.077 | 0.063 | 84.634*** |
| b=0.2 | 19 | 25.625 | 0.937 | -0.078 | 0.783 | -0.253 | 0.066 | 0.059 | 91.396*** |
| b=0.2 | 20 | 31.234 | 0.949 | -0.064 | 0.813 | -0.245 | 0.066 | 0.058 | 93.232*** |
| variable birth | 2 | 0.328 | 0.036 | -0.153 | -0.837 | NA | 0.000 | - | - |
| variable birth | 3 | 0.547 | 0.095 | -0.294 | -0.315 | -0.299 | 0.004 | - | - |
| variable birth | 4 | 0.809 | 0.168 | -0.235 | 1.369 | -0.894 | 0.035 | - | - |
| variable birth | 5 | 1.081 | 0.246 | -0.262 | 0.984 | -0.696 | 0.033 | 0.167 | 22.841*** |
| variable birth | 6 | 1.499 | 0.342 | -0.233 | 0.620 | -0.472 | 0.023 | 0.130 | 33.398*** |
| variable birth | 7 | 1.921 | 0.418 | -0.233 | 0.899 | -0.559 | 0.035 | 0.156 | 25.446*** |
| variable birth | 8 | 2.447 | 0.495 | -0.227 | 0.848 | -0.530 | 0.040 | 0.161 | 24.216*** |
| variable birth | 9 | 3.166 | 0.568 | -0.211 | 0.434 | -0.330 | 0.022 | 0.123 | 36.123*** |
| variable birth | 10 | 3.985 | 0.629 | -0.205 | 0.744 | -0.417 | 0.046 | 0.127 | 34.529*** |
| variable birth | 11 | 4.975 | 0.687 | -0.198 | 0.777 | -0.402 | 0.052 | 0.114 | 40.125*** |
| variable birth | 12 | 6.270 | 0.739 | -0.196 | 0.762 | -0.372 | 0.051 | 0.102 | 46.573*** |
| variable birth | 13 | 7.501 | 0.772 | -0.152 | 0.657 | -0.307 | 0.043 | 0.088 | 56.336*** |
| variable birth | 14 | 9.267 | 0.805 | -0.138 | 0.682 | -0.289 | 0.044 | 0.080 | 63.460*** |
| variable birth | 15 | 11.158 | 0.835 | -0.120 | 0.639 | -0.258 | 0.040 | 0.082 | 61.548*** |
| variable birth | 16 | 13.641 | 0.854 | -0.124 | 0.550 | -0.219 | 0.035 | 0.078 | 65.470*** |
| variable birth | 17 | 17.178 | 0.887 | -0.084 | 0.702 | -0.240 | 0.049 | 0.064 | 83.076*** |
| variable birth | 18 | 21.077 | 0.900 | -0.092 | 0.706 | -0.230 | 0.047 | 0.067 | 78.682*** |
| variable birth | 19 | 25.681 | 0.916 | -0.078 | 0.691 | -0.213 | 0.048 | 0.065 | 81.566*** |
| variable birth | 20 | 31.736 | 0.930 | -0.079 | 0.613 | -0.183 | 0.040 | 0.061 | 87.904*** |
| b=0.2, d=0.05 | 2 | 0.369 | 0.038 | -0.161 | 0.024 | -0.153 | 0.001 | - | - |
| b=0.2, d=0.05 | 3 | 0.588 | 0.094 | -0.292 | 0.240 | -0.408 | 0.012 | - | - |
| b=0.2, d=0.05 | 4 | 0.809 | 0.155 | -0.221 | 0.340 | -0.411 | 0.016 | - | - |
| b=0.2, d=0.05 | 5 | 1.076 | 0.234 | -0.187 | 0.338 | -0.368 | 0.018 | - | - |
| b=0.2, d=0.05 | 6 | 1.373 | 0.307 | -0.220 | 0.426 | -0.425 | 0.030 | 0.296 | 7.645** |
| b=0.2, d=0.05 | 7 | 1.695 | 0.379 | -0.194 | 0.541 | -0.460 | 0.041 | 0.143 | 29.054*** |
| b=0.2, d=0.05 | 8 | 2.066 | 0.448 | -0.167 | 0.379 | -0.328 | 0.026 | 0.131 | 33.033*** |
| b=0.2, d=0.05 | 9 | 2.521 | 0.514 | -0.136 | 0.451 | -0.333 | 0.033 | 0.133 | 32.319*** |
| b=0.2, d=0.05 | 10 | 3.052 | 0.572 | -0.128 | 0.390 | -0.278 | 0.027 | 0.097 | 49.734*** |
| b=0.2, d=0.05 | 11 | 3.595 | 0.625 | -0.078 | 0.459 | -0.276 | 0.029 | 0.106 | 44.260*** |
| b=0.2, d=0.05 | 12 | 4.225 | 0.666 | -0.059 | 0.433 | -0.239 | 0.025 | 0.082 | 61.548*** |
| b=0.2, d=0.05 | 13 | 4.912 | 0.705 | -0.035 | 0.390 | -0.198 | 0.019 | 0.068 | 77.304*** |
| b=0.2, d=0.05 | 14 | 5.992 | 0.753 | -0.036 | 0.458 | -0.217 | 0.026 | 0.073 | 70.980*** |
| b=0.2, d=0.05 | 15 | 6.874 | 0.777 | 0.029 | 0.511 | -0.203 | 0.026 | 0.050 | 110.574*** |
| b=0.2, d=0.05 | 16 | 7.999 | 0.805 | 0.062 | 0.415 | -0.142 | 0.014 | 0.048 | 115.814*** |
| b=0.2, d=0.05 | 17 | 9.457 | 0.833 | 0.091 | 0.472 | -0.146 | 0.017 | 0.041 | 138.188*** |
| b=0.2, d=0.05 | 18 | 11.110 | 0.860 | 0.137 | 0.463 | -0.120 | 0.012 | 0.030 | 194.468*** |
| b=0.2, d=0.05 | 19 | 12.731 | 0.873 | 0.167 | 0.514 | -0.122 | 0.013 | 0.029 | 201.703*** |
| b=0.2, d=0.05 | 20 | 14.820 | 0.892 | 0.226 | 0.474 | -0.084 | 0.007 | 0.023 | 258.331*** |
| b=0.2, d=0.2 | 2 | 0.419 | 0.337 | -0.084 | 0.389 | -0.416 | 0.006 | - | - |
| b=0.2, d=0.2 | 3 | 0.549 | 0.067 | -0.105 | 0.084 | -0.151 | 0.001 | - | - |
| b=0.2, d=0.2 | 4 | 0.673 | 0.108 | -0.138 | 0.182 | -0.251 | 0.004 | - | - |
| b=0.2, d=0.2 | 5 | 0.813 | 0.146 | -0.093 | 0.281 | -0.280 | 0.007 | - | - |
| b=0.2, d=0.2 | 6 | 0.904 | 0.182 | -0.095 | 0.253 | -0.255 | 0.007 | - | - |
| b=0.2, d=0.2 | 7 | 1.005 | 0.213 | -0.074 | 0.139 | -0.150 | 0.003 | - | - |
| b=0.2, d=0.2 | 8 | 1.109 | 0.241 | -0.036 | 0.032 | -0.046 | 0.000 | - | - |
| b=0.2, d=0.2 | 9 | 1.246 | 0.277 | 0.078 | 0.234 | -0.105 | 0.002 | - | - |
| b=0.2, d=0.2 | 10 | 1.325 | 0.303 | 0.057 | 0.014 | 0.028 | 0.000 | 0.037 | 154.779*** |
| b=0.2, d=0.2 | 11 | 1.416 | 0.327 | 0.094 | 0.153 | -0.038 | 0.000 | 0.071 | 73.402*** |
| b=0.2, d=0.2 | 12 | 1.575 | 0.358 | 0.145 | 0.173 | -0.018 | 0.000 | 0.025 | 236.434*** |
| b=0.2, d=0.2 | 13 | 1.674 | 0.376 | 0.178 | 0.006 | 0.107 | 0.002 | 0.061 | 87.904*** |
| b=0.2, d=0.2 | 14 | 1.821 | 0.402 | 0.202 | 0.030 | 0.105 | 0.002 | 0.028 | 209.455*** |
| b=0.2, d=0.2 | 15 | 1.921 | 0.418 | 0.243 | 0.089 | 0.092 | 0.002 | 0.015 | 404.334*** |
| b=0.2, d=0.2 | 16 | 2.007 | 0.439 | 0.260 | -0.054 | 0.187 | 0.009 | 0.005 | 1243.982*** |
| b=0.2, d=0.2 | 17 | 2.183 | 0.464 | 0.323 | -0.018 | 0.199 | 0.010 | 0.009 | 684.207*** |
| b=0.2, d=0.2 | 18 | 2.264 | 0.482 | 0.343 | -0.104 | 0.257 | 0.018 | 0.004 | 1558.859*** |
| b=0.2, d=0.2 | 19 | 2.344 | 0.490 | 0.353 | -0.083 | 0.248 | 0.018 | 0.010 | 614.237*** |
| b=0.2, d=0.2 | 20 | 2.525 | 0.518 | 0.403 | -0.134 | 0.301 | 0.027 | 0.011 | 556.990*** |

^†^The models used were (i) the pure birth model [1] with a per lineage birth rate of 0.2 per unit time duration, (ii) the pure birth model with the per lineage birth rate allowed to vary among (but not within) clades with a mean of 0.2 and standard deviation of 0.04, (iii) the birth-death model [2] with a per lineage birth rate of 0.2 and death rate of 0.05 per unit time and (iv) the birth-death model with both birth and death rates equal to 0.2.

^‡^For each simulation duration 10,000 replicates were run. If the entire clade was extinct prior to the end of the simulation duration then this run would be repeated.

^§^Average clade size was estimated as the mean of the log transformed clade size data (where a lineage that has not split has a clade size of zero) plus 1. The reported clade sizes have been back transformed using the natural exponent minus 1.

^¶^ The γ statistic of Pybus and Harvey [3] was only calculated in cases where clade size was ≥ 3.

^††^The intercept, slope and R^2^ reported are all derived from a linear regression of γ on clade size (log transformed).

^‡‡^The proportion of simulated trees with > 15 lineages that were found to show significant slowdowns (corrected γ < -1.645, P < 0.05).

^§§^χ^2^ statistic derived from a goodness of fit test (with d.f. = 1 and applying Yates’ continuity correction where expected frequency < 5) comparing the observed frequency of significant slowdowns in trees of size > 15 to the distribution expected under a constant rate model. *, **, and *** denote significance at the *P* < 0.05, 0.01, and 0.001 levels, respectively. In simulations where < 20 clades possessed > 15 lineages the test was not conducted as the expected frequencies could not be estimated with confidence. In some cases the χ^2^ statistic could not be calculated due to expected frequencies of zero.

# References

1. Yule GU (1925) A mathematical theory of evolution, based on the conclusions of Dr. J. C. Willis, F.R.S. Philosophical Transactions of the Royal Society B 213: 21-87.

2. Raup DM, Gould SJ, Schopf TJM, Simberloff DS (1973) Stochastic models of phylogeny and the evolution of diversity. Journal of Geology 81: 525-542.

3. Pybus OG, Harvey PH (2000) Testing macro-evolutionary models using incomplete molecular phylogenies. Proc R Soc Lond B 267: 2267-2272.
