# Supplementary material for: Density-Dependent Cladogenesis in Birds
Source: PLoS Biol. 2008 Mar 25;6(3):e71. doi: 10.1371/journal.pbio.0060071 (PMC2270327; doi:10.1371/journal.pbio.0060071)
Supplement: Table S2 — (132 KB DOC) [file pbio.0060071.st002.doc]

Table S2. Major sources of phylogenetic information.

| Phylogeny | Genes | Source |
| --- | --- | --- |
| wrens | cytochrome *b* | [1,2] |
| *Phylloscopus* & *Seicercus* | cytochrome *b* | [3,4] |
| *Anthus* | cytochrome *b* | [5] |
| *Catharus* | cytochrome *b* | [6,7] |
| grackles & allies | cytochrome *b*, ND2 | [8] |
| Estrildidae | ND2, ND6 | [9] * |
| *Parus* | cytochrome *b* | [10] |
| *Tangara* | cytochrome *b*, ND2 | [11] |
| *Turdus* & allies | cytochrome *b*, ND2, ND3 | [12] |
| *Dendroica*, *Parula*, *Seiurus*, *Vermivora* | cytochrome *b*, ND2, ATPase 6, ATPase 8 | [13-15] |
| *Amazona* | COI | [16] |
| *Tringa* | cytochrome *b*, ND2 | [17] |
| swallows | cytochrome *b*, ND3 | [18,19] |
| caciques & oropendolas | cytochrome *b* | [20,21] |
| *Ficedula* | cytochrome *b*, ND2 | [22] |
| *Hemispingus* | ND2 | [23] |
| *Acanthiza* | cytochrome *b* | [24] |
| *Anas* | cytochrome *b* | [25,26] |
| *Toxostoma* | cytochrome *b*, ND2 | [27] |
| *Thamnophilus* | cytochrome *b*, ND2, ND3 | [28] |
| *Geositta* | cytochrome *b*, ND2, ND3 | [29] |
| storks | cytochrome *b* | [30] |
| *Meliphaga* | cytochrome *b* | [31] |
| Trogons | cytochrome *b*, ND2 | [32] |
| *Sylvia* | cytochrome *b* | [33] |
| Alcinae | cytochrome *b* | [34] |
| *Picoides* & *Veniliornis* | cytochrome *b*, COI | [35,36] |
| *Empidonax* | cytochrome *b*, ND2, ND3 | [37] |
| *Icterus* | cytochrome *b*, ND2 | [38] |
| *Crax* | ND2 | [39] |
| *Ramphastos* | cytochrome *b*, ND2 | [40] |
| *Aegotheles* | cytochrome *b* | [41] |
| penguins | cytochrome *b*, COI | [42] |
| *Pteroglossus* | cytochrome *b,* ATPase 6 | [43] |
| *Larus* | cytochrome *b* | [44] |
| grouse, turkeys, partridges & tragopans | cytochrome *b*, ND2 | [45-47] |
| *Myioborus* | cytochrome *b* | [48] |
| *Alectoris* | cytochrome *b* | [49] |
| *Cinclodes* | COI, ND3 | [50] |
| cranes | cytochrome *b* | [51] |
| albatrosses | cytochrome *b* | [52] |
| *Sterna* | cytochrome *b*, ND2 | [53] |
| *Puffinus* | cytochrome *b* | [54] |
| *Tauraco* | cytochrome | [55] |
| *Myiarchus* | ATPase 6, ATPase 8 | [56] |

* in addition unpublished sequence data was provided by M. Sorenson and R. Payne.

**References**

1. Barker FK (2007) Avifaunal interchange across the Panamanian isthmus: insights from *Campylorhynchus* wrens. Biol JLinn Soc 90: 687-702.

2. Mann NI, Barker FK, Graves JA, Dingess-Mann KA, Slater PJB (2006) Molecular data delineate four genera of "*Thryothorus*" wrens. Mol Phyl Evol 40: 750-759.

3. Olsson U, Alström P, Ericson PGP, Sundberg P (2005) Non-monophyletic taxa and cryptic species—Evidence from a molecular phylogeny of leaf-warblers (*Phylloscopus*, Aves). Mol Phyl Evol 36: 261-276.

4. Olsson U, Alström P, Sundberg P (2004) Non-monophyly of the avian genus Seicercus (Aves: Sylviidae) revealed by mitochondrial DNA. Zool Scr 33: 501-510.

5. Voelker G (1999) Molecular evolutionary relationships in the avian genus Anthus (Pipits: Motacillidae). Mol Phyl Evol 11: 84-94.

6. Outlaw DC, Voelker G, Boria M, Girman DJ (2003) Evolution of long-distance migration in and historical biogeography of *Catharus* thrushes: a molecular phylogenetic approach. Auk 120: 299-310.

7. Winker K, Pruett CL (2006) Seasonal migration, speciation, and morphological convergence in the *Catharus* thrushes (Aves: Turdidae). Auk 123: 1052-1068.

8. Johnson KP, Lanyon SM (1999) Molecular systematics of the grackles and allies, and the effect of additional sequence (cyt b and ND2). Auk 116: 759-768.

9. Sorenson MD, Sefc KM, Payne RB (2003) Speciation by host switch in brood parasitic indigobirds. Nature 424: 928-931.

10. Gill FB, Slikas B, Sheldon FH (2005) Phylogeny of titmice (Paridae): II. Species relationships based on sequences of the mitochondrial cytochrome-b gene. J Evol Biol 122: 121-143.

11. Burns KJ, Naoki K (2004) Molecular phylogenetics and biogeography of Neotropical tanagers in the genus Tangara. Mol Phyl Evol 32: 838-854.

12. Voelker G, Rohwer S, Bowie RCK, Outlaw DC (2007) Molecular systematics of a speciose, cosmopolitan songbird genus: Defining the limits of, and relationships among, the *Turdus* thrushes. Mol Phyl Evol 42: 422-434.

13. Lovette IJ, Bermingham E (1999) Explosive speciation in the New World *Dendroica* warblers. Proc R Soc Lond B 266: 1629-1636.

14. Lovette IJ, Bermingham E (2002) What is a wood-warbler? Molecular characterization of a monophyletic Parulidae. Auk 119: 965-714.

15. Lovette IJ, Bermingham E (2001) Mitochondrial perspective on the phylogenetic relationships of the *Parula* wood-warblers. Auk 118: 211-215.

16. Russello MA, Amato G (2004) A molecular phylogeny of Amazona: implications for Neotropical parrot biogeography, taxonomy, and conservation. Mol Phyl Evol 30: 421-437.

17. Pereira SL, Baker AJ (2005) Multiple gene evidence for parallel evolution and retention of ancestral morphological states in the shanks (Charadriiformes: Scolopacidae). Condor 107: 514-526.

18. Sheldon FH, Whittingham LA, Moyle RG, Slikas B, Winkler DW (2005) Phylogeny of swallows (Aves: Hirundinidae) estimated from nuclear and mitochondrial DNA sequences. Molecular Phylogenetics and Evolution 35: 254-270.

19. Whittingham LA, Slikas B, Winkler DW, Sheldon FH (2002) Phylogeny of the Tree Swallow Genus, *Tachycineta* (Aves: Hirundinidae), by Bayesian Analysis of Mitochondrial DNA Sequences. Mol Phyl Evol 22: 430-441.

20. Price JJ, Lanyon SM (2002) A robust phylogeny of the oropendolas: polyphyly revealed by mitochondrial sequence data. Auk 119: 335-348.

21. Lanyon SM, Omland KE (1999) A molecular phylogeny of the blackbirds (Icteridae): Five lineages revealed by cytochrome-*b* sequence data. Auk 116: 629-639.

22. Outlaw DC, Voelker G (2006) Systematics of *Ficedula* flycatchers (Muscicapidae): A molecular reassessment of a taxonomic enigma. 41.

23. Garcia-Moreno J, Ohlson J, Fjeldsa J (2001) MtDNA sequences support monophyly of *Hemispingus* tanagers. Mol Phylo Evol 21: 424-435.

24. Nicholls JA (2001) Molecular systematics of the thornbills Acanthiza. Emu 101: 33-37.

25. Johnson KP, Sorenson MD (1999) Phylogeny and biogeography of dabbling ducks (Genus: *Anas*): A comparison of molecular and morphological evidence. Auk 116: 792-805.

26. Johnson KP, Sorenson MD (1998) Comparing Molecular Evolution in Two Mitochondrial Protein Coding Genes (Cytochromeband ND2) in the Dabbling Ducks (Tribe: Anatini). Molecular Phylogenetics and Evolution 10: 82-94.

27. Zink RM, Dittmann DL, Klicka J, Blackwell RC (1999) Evolutionary patterns of morphometrics, allozymes, and mitochondrial DNA in thrashers (Genus *Toxostoma*). Auk 116: 1021-1038.

28. Brumfield RT, Edwards SV (2007) Evolution into and out of the Andes: a Bayesian analysis of historical diversification in *Thamnophilus* antshrikes. Evolution 61: 346-367.

29. Cheviron ZA, Capparella AP, Vuilleumier F (2005) Molecular phylogenetic relationships among the *Geositta* miners (Furnariidae) and biogeographic implications for avian speciation in Fuego-Patagonia. Auk 122: 158-174.

30. Slikas B (1997) Phylogeny of the avian family Ciconiidae (Storks) based on cytochrome b sequences and DNA-DNA hybridization distances. Mol Phyl Evol 8: 275-300.

31. Norman JA, Rheindt FE, Rowe DL, Christidis L (2007) Speciation dynamics in the Australo-Papuan *Meliphaga* honeyeaters. Mol Phyl Evol 42: 80-91.

32. Moyle RG (2005) Phylogeny and biogeographical history of Trogoniformes, a pantropical bird order. Biol J Linn Soc 84: 725-738.

33. Böhning-Gaese K, Schuda MD, Helbig AJ (2003) Weak phylogenetic effects on ecological niches of Sylvia warblers. J Evol Biol 16: 956-965.

34. Moum T, Johansen S, Erikstad K, Piatt J (1994) Phylogeny and evolution of the auks (Subfamily Alcinae) based on mitochondrial DNA sequences. PNAS 91: 7912-7916.

35. Moore WS, Weibel AC, Agius A (2006) Mitochondrial DNA phylogeny of the woodpecker genus Veniliornis (Picidae, Picinae) and related genera implies convergent evolution of plumage patterns. Biol J Linn Soc 87: 611-624.

36. Weibel AC, Moore WS (2002) A Test of a Mitochondrial Gene-Based Phylogeny of Woodpeckers (Genus *Picoides*) Using an Independent Nuclear Gene, [beta]-Fibrinogen Intron 7. Mol Phyl Evol 22: 247-257.

37. Johnson NK, Cicero C (2002) The role of ecologic diversification in sibling speciation of *Empidonax* flycatchers (Tyrannidae): multigene evidence from mtDNA. Molecular Ecology 11: 2065-2081.

38. Omland KE, Lanyon SM, Fritz SJ (1999) A molecular phylogeny of the new world orioles (Icterus): The importance of dense taxon sampling. Mol Phyl Evol 12: 224-239.

39. Pereira SL, Baker AJ (2004) Vicariant speciation of curassows (Aves, Cracidae): A hypothesis based on mitochondrial DNA phylogeny. Auk 121: 682-694.

40. Weckstein JD (2005) Molecular phylogenetics of teh Ramphastos toucans: implications for the evolution of morphology, vocalizations, and coloration. Auk 122: 1191-1209.

41. Dumbacher JP, Pratt TK, Fleischer RC (2003) Phylogeny of the owlet-nightjars (Aves : Aegothelidae) based on mitochondrial DNA sequence. Mol Phyl Evol 29: 540-549.

42. Baker AJ, Pereira SL, Haddrath OP, Edge K-A (2006) Multiple gene evidence for expansion of extant penguins out of Antarctica due to global cooling. Proc R Soc Lond B 273: 11-17.

43. Eberhard JR, Bermingham E (2005) Phylogeny and comparative biogeography of *Pionopsitta* parrots and *Pteroglossus* toucans. Mol Phyl Evol 36: 288-304.

44. Pons J-M, Hassanin A, Crochet P-A (2005) Phylogenetic relationships within the Laridae (Charadriiformes: Aves) inferred from mitochondrial markers. Mol Phyl Evol 37: 686-699.

45. Dimcheff DE, Drovetski SV, Mindell DP (2002) Molecular evolution and systematics of Tetraoninae and other Galliformes using mitochondrial 12S and ND2 genes. Mol Phyl Evol 24: 203-215.

46. Randi E, Lucchini V, Armijo-Prewitt T, Kimball RT, Braun EL, et al. (2000) Mitochondrial DNA phylogeny and speciation in the tragopans. Auk 117: 1003-1015.

47. Randi E, Lucchini V, Hennache A, Kimball RT, Braun EL, et al. (2001) Evolution of the mitochondrial DNA control region and cytochrome b genes and the inference of phylogenetic relationships in the avian genus *Lophura* (Galliformes). Mol Phylo Evol 19: 187-201.

48. Perez-Eman JL (2005) Molecular phylogenetics and biogeography of the Neotropical redstarts (*Myioborus*; Aves, Parulinae). Mol Phyl Evol 37: 511-528.

49. Randi E (1996) A mitochondrial cytochrome b phylogeny of the *Alectoris* partridges. Mol Phyl Evol 6: 214-227.

50. Chesser RT (2004) Systematics, evolution, and biogeography of the South American ovenbird genus Cinclodes. Auk 121: 752-766.

51. Krajewski C, Fetzner JW (1994) Phylogeny of cranes (Gruiformes: Gruidae) based on cytochrome-*b* DNA sequences. Auk 111: 351-365.

52. Nunn GB, Cooper J, Jouventin P, Robertson CJR, Robertson GG (1996) Evolutionary relationships among extant albatrosses (Procellariformes: Diomedeidae) established from complete cytochrome-b gene sequenes. Auk 113: 784-801.

53. Bridge ES, Jones AW, Baker AJ (2005) A phylogenetic framework for the terns (Sternini) inferred from mtDNA sequences: implications for taxonomy and plumage evolution. Mol Phyl Evol 35: 459-469.

54. Austin JJ, Bretagnolle V, Pasquet E (2004) A global molecular phylogeny of the small Puffinus shearwaters and implications for systematics of the little Audubon's shearwater complex. The Auk 121: 847-864.

55. Veron G, Winney BJ (2000) Phylogenetic relationships within the turacos (Musophagidae). Ibis 142: 446-456.

56. Joseph L, Wilke T, Bermingham E, Alpers D, Ricklefs R (2004) Towards a phylogenetic framework for the evolution of shakes, rattles, and rolls in *Myiarchus* tyrant-flycatchers (Aves: Passeriformes: Tyrannidae). Mol Phyl Evol 31: 139-152.
